# Association of *VAX1, MAFB, WNT3* with Non-Syndromic Cleft Lip with or without Cleft Palate in a Japanese Population

**DOI:** 10.3390/genes16080862

**Published:** 2025-07-24

**Authors:** Tran Phuong Thao, Teruyuki Niimi, Satoshi Suzuki, Toko Hayakawa, Chisato Sakuma, Ken Kitagawa, Hideto Imura, Hisataka Kondo, Nguyen Huu Tu, Tong Minh Son, Vo Truong Nhu Ngoc, Le Kha Anh, Pham Nguyen Gia Loc, Hiroo Furukawa, Nagana Natsume, Nagato Natsume

**Affiliations:** 1 Division of Research and Treatment for Oral and Maxillofacial Congenital Anomalies, Aichi Gakuin University, 2–11 Suemori-dori, Chikusa-ku, Nagoya 464-8651, Japan; tranphuongthao311099@gmail.com (T.P.T.); niimi@dpc.agu.ac.jp (T.N.); zsatoshi@gmail.com (S.S.); char_nyoko@yahoo.co.jp (C.S.); 9646ken@gmail.com (K.K.); h-imura@dpc.agu.ac.jp (H.I.); lekhaanh268@gmail.com (L.K.A.); gialocp@gmail.com (P.N.G.L.); triple.n.8924@gmail.com (N.N.); 2 School of Dentistry, Hanoi Medical University, Hanoi 10000, Vietnam; tongminhson@hmu.edu.vn (T.M.S.); nhungoc@hmu.edu.vn (V.T.N.N.); 3 Cleft Lip and Palate Center, Aichi Gakuin Dental Hospital, 2-11 Suemori-dori, Chikusa-ku, Nagoya 464-8651, Japan; hayakawa@dpc.agu.ac.jp; 4 Division of Speech, Hearing, and Language, Aichi Gakuin Dental Hospital, 2-11 Suemori-dori, Chikusa-ku, Nagoya 464-8651, Japan; hfuru@dpc.aichi-gakuin.ac.jp; 5 Junior College, Aichi Gakuin University, Nagoya 464-0037, Japan; kondoh@dpc.agu.ac.jp; 6 Department of Anesthesia—Resuscitation, Hanoi Medical University, Hanoi 10000, Vietnam; nguyenhuutu@hmu.edu.vn; 7 Odonto-Maxillo Facial Hospital of Ho Chi Minh City, 263-265 Tran Hung Dao Street, District 1, Ho Chi Minh City 71000, Vietnam

**Keywords:** cleft lip with or without palate, *VAX1*, *MAFB*, *WNT3*, Japanese population

## Abstract

**Background/Objectives:** Non-syndromic cleft lip with or without palate (NSCL/P) is a common, multifactorial congenital anomaly. As genetic associations can be population-specific, this study aimed to investigate single-nucleotide polymorphisms (SNPs) in the *VAX1*, *MAFB*, and *WNT3* genes for association with NSCL/P in a Japanese cohort. **Methods:** A case–control study was conducted with 310 Japanese patients with NSCL/P and 308 ethnically matched healthy controls from Aichi Gakuin Dental Hospital. We genotyped SNPs rs7078160 (*VAX1*), rs13041247 (*MAFB*), and rs3809857 (*WNT3*) using TaqMan assays. Associations were assessed using chi-squared tests, with results stratified by sex and corrected for multiple comparisons using the Bonferroni method. **Results:** The *VAX1* rs7078160 A allele was significantly associated with an increased risk for NSCL/P (OR = 1.67, *p* < 0.00001). The association was particularly strong in females (OR = 1.93, *p* < 0.00001) but not significant in males after correction. The *MAFB* rs13041247 variant showed a nominal protective association with the NSCLO subtype that was not significant after Bonferroni correction. No significant association was found for *WNT3*. A notable gene–gene interaction was observed, where carrying risk alleles for both *VAX1* and *MAFB* significantly increased overall NSCL/P risk (OR = 2.65, *p* = 0.00008). **Conclusions:** *VAX1* rs7078160 is a significant risk factor for NSCL/P in the Japanese population, with a pronounced female-specific effect. A synergistic interaction between *VAX1* and *MAFB* elevates disease risk, whereas *WNT3* was not implicated in this cohort. These findings underscore the population-specific genetic architecture of NSCL/P.

## 1. Introduction

Non-syndromic cleft lip with or without cleft palate (NSCL/P) is among the most common congenital malformations worldwide, with a global prevalence of 1–2 per 1000 live births. In Asian populations, the prevalence is approximately 1.19 per 1000, with a similar rate of 1.18 per 1000 in Japanese cohorts [1]. NSCL/P significantly impacts speech, feeding, and appearance, leading to substantial functional and cosmetic challenges. These issues often result in delayed development, impaired social integration, and a reduced quality of life. Despite advancements in surgical interventions and rehabilitation, NSCL/P remains a considerable burden on affected individuals, their families, and society.

The etiology is multifactorial, involving both genetic and environmental factors [2]. Identified environmental risk factors include maternal smoking, alcohol consumption, certain medications, and nutritional deficiencies [3]. Nevertheless, classic experimental studies in mice have underscored the fundamental importance of genetics, demonstrating that an individual’s genetic predisposition can override the maternal uterine environment in cleft development [4,5]. This strong genetic determinism provides the rationale for large-scale genetic investigations. Genome-wide association studies (GWASs) have identified various candidate genes implicated in NSCL/P development. Beaty et al. (2010) reported that the V-Maf musculoaponeurotic fibrosarcoma oncogene homolog B (*MAFB*) gene on 20q.12 was associated with genome-wide significance with NSCL/P [6]. *MAFB* encodes a transcription factor essential for developing the hindbrain, the thymus, interneurons, pancreatic islet cells, and the hematopoietic system [7]. Its expression pattern in mice supports its role in lip and palate formation [7]. Subsequent meta-analyses and population studies in Chinese, Vietnamese, and Colombian cohorts further corroborated the involvement of *MAFB* in NSCL/P [8,9,10,11].

The Ventral anterior homeobox 1 (*VAX1*) gene, on chromosome 10q25.3, encodes a transcription factor with a conserved homeodomain DNA-binding motif critical for embryonic development [12]. *VAX1* is involved in the formation of craniofacial structures, including the eyes, nose, and upper jaw. *VAX1* deficiency in murine models leads to abnormal craniofacial development, which can include the formation of a cleft palate [13]. rs7078160 at 10q25 (*VAX1*) was among the most significant SNPs in the German case–control GWAS and achieved genome-wide significance in the NSCL/P group in a European case-parent trio [14]. Beaty also reported that rs7078160 at *VAX1* was genome-wide significant in NSCL/P, with these findings replicated across several Asia populations [15,16,17].

The *WNT* gene family comprises members such as *WNT3*, *WNT3A*, *WNT5A*, *WNT8A*, and *WNT11*, which encode conserved secreted glycoproteins critical for developmental and cellular processes, including craniofacial embryogenesis [18,19,20]. Alterations within the *WNT* signaling pathway, encompassing both *WNT* genes and their downstream effectors, are associated with the risk of human NSCL/P [21]. Among them, *WNT3*, located on chromosome 17q21, has emerged as a prominent candidate gene for NSCL/P, though findings on its association across populations have been inconsistent [22,23,24].

This study used case–control approaches to investigate the associations of the *MAFB* gene, *VAX1* gene, and *WNT3* gene with NSCL/P and its subtypes in a Japanese population. By elucidating the genetic underpinnings of NSCL/P, this work aims to contribute to a deeper understanding of its etiology and facilitate the development of targeted interventions.

## 2. Materials and Methods

### 2.1. Subjects

The study consisted of 310 NSCL/P patients, including non-syndromic cleft lip only (NSCLO) and non-syndromic cleft lip and palate (NSCLP), and ethnically matched 308 region-matched healthy controls with no cleft history in their families (Table 1). All affected individuals in this study were diagnosed with NSCL/P following a strict and comprehensive clinical evaluation at the Aichi Gakuin Dental Hospital, Aichi, Japan. The diagnostic process followed standard clinical protocols and included a review of surgical history and a detailed examination of both extraoral and intraoral structures by experienced clinicians. This rigorous assessment was essential to prevent misclassification in cases with subtle or surgically corrected syndromic features (e.g., blepharophimosis or syngnathia) that might not be apparent during routine screening [25].

All participants identified as Japanese; provided their name, gender, and age; and were recruited from Aichi Gakuin Dental Hospital in Aichi, Japan. Written informed consent was obtained from all participants prior to enrollment. For individuals under 18 years of age, consent was secured from a parent or legal guardian. Peripheral blood samples were used for blood tests and stored at World Cleft Gene Banking in Aichi Gakuin University, Nagoya, Japan.

The estimated sample size, power, and effect size were calculated a priori using G*Power software (version 3.1.9.7), based on the assumption of a 1:1 case–control ratio and a significance level of 5% in order to ensure 80% statistical power.

This study was conducted in full accordance with the ethical principles of the Declaration of Helsinki (World Medical Association 2013). The research protocol was reviewed and approved by the Ethics Committee of Aichi Gakuin University (Approval No. 689) on 14 December 2023.

### 2.2. Genetic Analysis Procedures

Three SNPs (*MAFB* rs13041247, *VAX1* rs7078160, and *WNT3* rs3809857) were selected for genotyping based on previous GWASs and association studies, and the minor allele frequency (MAF) of the Asian population above 5% from the 1000 Genomes database [26].

Following the manufacturer’s protocol, DNA was extracted from fresh blood using NucleoSpin Tissue Genomic DNA Purification (MACHEREY-NAGEL, Düren, Germany). Spectrophotometric tests confirmed the purity of the DNA. Genotyping was performed with the standardized and experimentally validated TaqMan SNP genotyping assay (Applied Biosystems, Foster City, CA, USA) in the StepOnePlus Real-Time PCR System (Applied Biosystems, Foster City, CA, USA). For quality control, a minimum genotype call rate of 98% was required for inclusion.

### 2.3. Statistical Analysis

Hardy–Weinberg equilibrium (HWE) was evaluated for each SNP in the healthy control group using the chi-squared test. Differences in genotype and allele frequencies between the case and control groups were assessed using chi-squared or Fisher’s exact tests. To estimate the strength of association, odds ratios (ORs) and their corresponding 95% confidence intervals (CIs) were calculated. The primary association analysis was conducted under dominant and recessive genetic models. In the dominant model, individuals homozygous for the major allele were compared against a combined group of those with heterozygous and minor allele homozygous genotypes. Conversely, for the recessive model, the comparison was made between individuals homozygous for the minor allele and a combined group of major allele homozygotes and heterozygotes. This approach was chosen for several reasons: it provides a clear biological interpretation of risk based on the number of risk alleles, it is a standard method when the precise mode of inheritance is unknown, it facilitates direct comparison with a wide range of previous studies in non-syndromic cleft lip with or without palate (NSCL/P) genetics, and it limits the total number of statistical tests performed, thereby mitigating the risk of Type I errors. We used a Bonferroni correction to adjust for multiple comparisons, establishing a significance threshold of *p* ≤ 0.008 for the 6 primary tests in the case–control study (3 SNPs × 2 phenotypic groups) and *p* ≤ 0.004 for the association studies in males and females (3 SNPs × 2 phenotypic groups × 2 genders). For the gene–gene interaction analysis, which assessed three genetic risk groups (*VAX1*, *MAFB*, and both *VAX1-MAFB*) across two phenotypic subgroups (NSCLO and NSCLP), the significance threshold was conservatively set at *p* ≤ 0.008 (0.05/6 tests). Additionally, post hoc power analyses were conducted using G*Power (version 3.1.9.7) with a two-tailed test, an adjusted α of 0.008, and the actual sample size of 618 (310 cases and 308 controls) to determine detectable odds ratios (ORs) across minor allele frequencies (MAFs) from Table 2. The results are presented in Supplementary Table S1.

## 3. Results

The genotype frequencies for all three SNPs in the control group were in Hardy–Weinberg equilibrium (*p* > 0.05) (Table 2).

### 3.1. Case–Control Comparisons

#### 3.1.1. Overall Population

**Figure 1:**
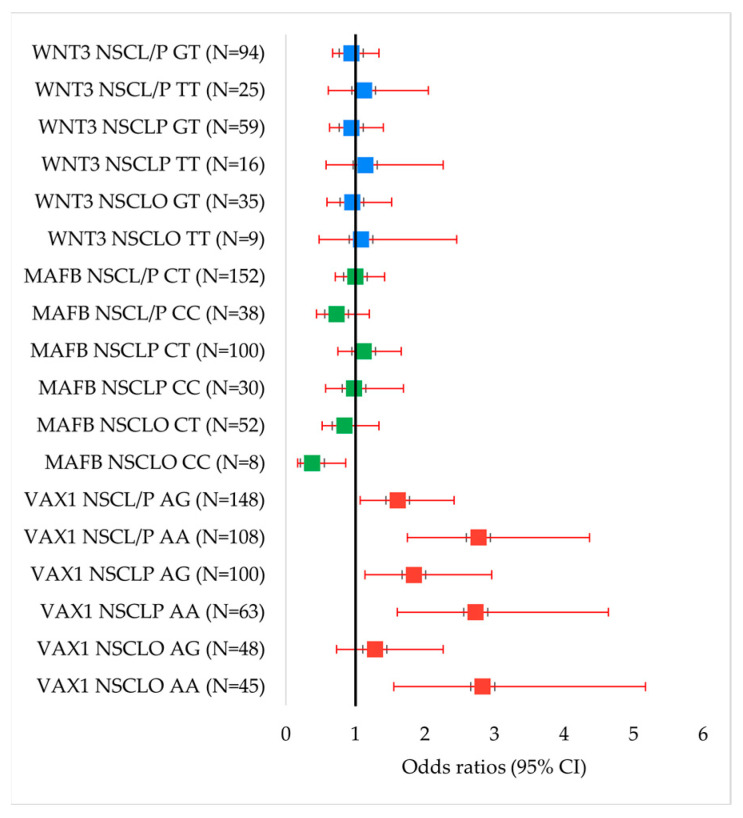
Forest plots of odds ratios (ORs) and 95% confidence intervals (CIs) for the associations between genotypes of *VAX1* rs7078160, *MAFB* rs13041247, and *WNT3* rs3809857 with NSCL/P and its subtypes. Significant associations were observed for *VAX1* genotypes.

The associations between the three selected SNPs and the risk of NSCL/P, as well as its subtypes (NSCLO and NSCLP), are presented in Table 3. A forest plot (Figure 1) was constructed to visually summarize the odds ratios and 95% confidence intervals for the genotype associations of *VAX1*, *MAFB*, and *WNT3* with NSCL/P and its subtypes.

***VAX1* rs7078160**

The *VAX1* rs7078160 polymorphism revealed significant associations. In the NSCL/P group, genotype AA and allele A were significantly different from those among the controls (*p* values were < 0.00001). The odds ratios (ORs) were 2.77 (95% CI = 1.75–4.37) for genotype AA and 1.67 (95% CI = 1.34–2.1) for allele A, respectively. Genotype analyses identified the association of *VAX1* rs7078160 under the dominant model (OR = 1.96, 95% CI = 1.33–2.87, and *p* = 0.0005).

For the NSCLP subtype, the *VAX1* rs 7078160 polymorphism showed statistically significant differences in genotype AA and allele A compared to the control group (*p* values of 0.0002). The odds ratios (ORs) were 2.73 (95% CI = 1.60–4.64) for genotype AA and 1.62 (95% CI = 1.26–2.09) for allele A, respectively. When examining the genotype model, the dominant and recessive model analysis further confirmed the association, showing odds ratios of 2.10 (95% CI = 1.34–3.30, and *p* = 0.0011) and 1.78 (95% CI = 1.19–2.68, and *p* = 0.005), underscoring the potential genetic influence on NSCLP susceptibility.

Within the NSCLO subgroup, both the A allele (OR = 1.77, *p* = 0.0003) and the AA genotype (OR = 2.83, *p* = 0.0006) were significantly associated with increased risk. This was further supported by a recessive model, which demonstrated that the AA genotype was associated with 2.40-fold increased odds of NSCLO compared to the combined GG + AG genotypes (*p* = 0.0002).

***MAFB* rs13041247**

In the overall population analysis with a conventional significance threshold (*p* < 0.05), the *MAFB* rs13041247 polymorphism showed significant associations with NSCLO. The homozygous CC genotype demonstrated a protective effect against NSCLO (OR = 0.38, 95% CI: 0.17–0.86, *p* = 0.017). In contrast, the recessive model (CC/TT + CT) was significantly associated with increased risk of NSCLO (OR = 0.42, 95% CI: 0.19–0.91, *p* = 0.024). At the allelic level, the C allele showed a protective effect against NSCLO (OR = 0.70, 95% CI: 0.50–0.97, *p* = 0.032).

No significant associations were found between *MAFB* rs13041247 and NSCLP or overall NSCL/P at the conventional significance threshold (*p* < 0.05). After applying the Bonferroni adjustment for multiple testing (*p* < 0.008), none of the associations with *MAFB* rs13041247 remained statistically significant.

***WNT3* rs3809857**

There was no evidence of genotypic or allele association with the Japanese population’s susceptibility to NSCL/P, NSCLP, and NSCLO for *WNT3* rs3809857.

#### 3.1.2. Sex-Stratified Analysis Results

In the sex-stratified analysis with Bonferroni-adjusted significance threshold (*p* < 0.004), significant associations were observed between *VAX1* rs7078160 and NSCL/P in females but not in males (Table 4).

**Figure 2:**
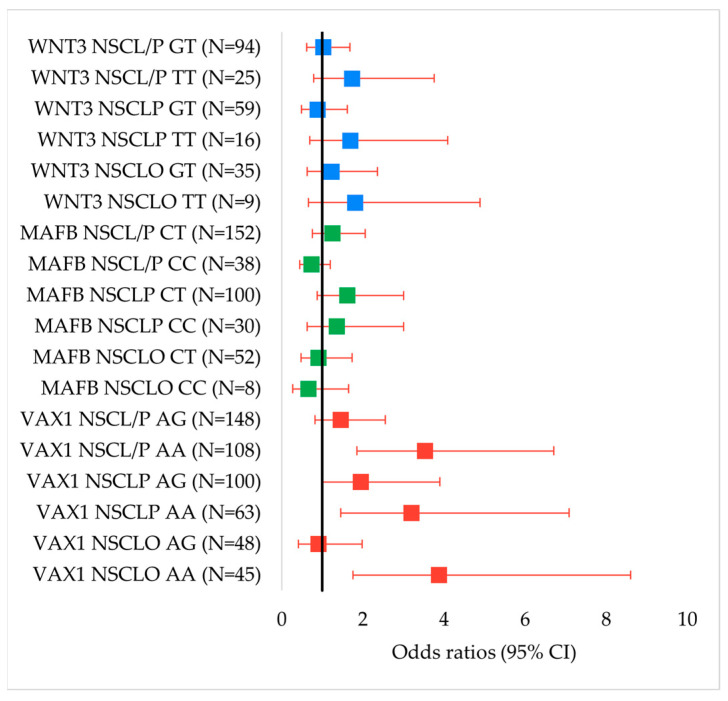
Forest plots of odds ratios (ORs) and 95% confidence intervals (CIs) for the associations between genotypes of *VAX1* rs7078160, *MAFB* rs13041247, and *WNT3* rs3809857 with NSCL/P and its subtypes in females. Significant associations were observed for *VAX1* genotypes.

Among females, the homozygous AA genotype showed significant associations with increased risk of NSCLO (OR = 3.88, 95% CI: 1.75–8.60, *p* = 0.0006), NSCLP (OR = 3.2, 95% CI: 1.45–7.08, *p* = 0.0034), and overall NSCL/P (OR = 3.53, 95% CI: 1.85–6.71, *p* < 0.00001), all meeting the Bonferroni-adjusted significance threshold. The recessive model (AA/GG + AG) showed significant protective effects for NSCLO (OR = 4.12, 95% CI: 2.15–7.89, *p* < 0.00001) and NSCL/P (OR = 2.77, 95% CI: 1.64–4.69, *p* = 0.0001). At the allelic level, the A allele was significantly associated with NSCLO (OR = 2.22, 95% CI: 1.44–3.42, *p* = 0.0003), NSCLP (OR = 1.74, 95% CI: 1.19–2.55, *p* = 0.0038), and NSCL/P (OR = 1.93, 95% CI: 1.40–2.66, *p* < 0.00001) (Figure 2).

**Figure 3:**
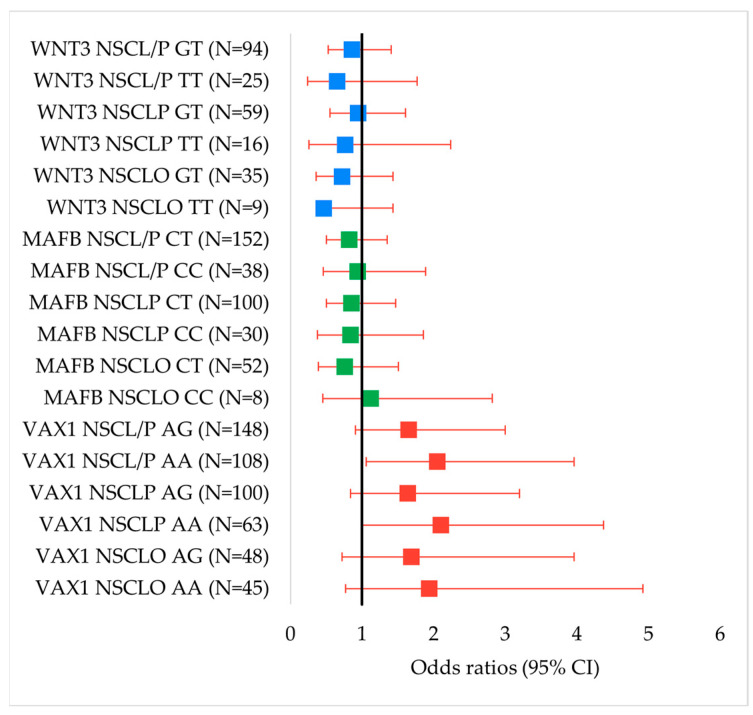
Forest plots of odds ratios (ORs) and 95% confidence intervals (CIs) for the associations between genotypes of *VAX1* rs7078160, *MAFB* rs13041247, and *WNT3* rs3809857 with NSCL/P and its subtypes in males.

In males, no significant associations were observed between *VAX1* rs7078160 genotypes or alleles and any type of NSCL/P (Figure 3).

For the *MAFB* rs13041247 and *WNT3* rs3809857 polymorphism, no significant associations were detected with any type of NSCL/P in either females or males across genotypic, allelic, dominant, or recessive genetic models with or without applying the Bonferroni correction.

### 3.2. Gene–Gene Interaction

Building upon the strong association observed for *VAX1* and the weaker association for *MAFB*, we investigated their potential synergistic interaction. To test whether the presence of a second risk variant would amplify disease susceptibility, we compared individuals carrying risk alleles at both loci (*VAX1* rs7078160-A and *MAFB* rs13041247-T) against those with single-locus risk. As shown in Table 5, individuals carrying risk alleles for both genes demonstrated a nearly threefold increase in disease risk compared to those carrying only the *MAFB* risk allele.

## 4. Discussion

The genetic etiology of NSCL/P is known to have population-specific characteristics, necessitating focused research within distinct ethnic groups. In the Japanese population, previous association studies have provided valuable insights, identifying risk variants in genes such as *PAX9*, *TGFB3*, *GAD67*, *DLX4*, and *PAX7* [15,27,28,29]. Despite their prominence as candidate genes in global populations, association studies for *MAFB* and *WNT3* have yet to be conducted exclusively within a Japanese cohort. Furthermore, while a key study implicated *VAX1* in an Asian population, that analysis included both Japanese and Mongolian subjects, which could obscure population-specific genetic effects due to ethnic admixture [15]. Therefore, the present study was designed to address these specific gaps by assessing the associations of *MAFB*, *WNT3*, and *VAX1* in a large, homogenous Japanese population, aiming to provide a more precise, population-specific risk profile.

The ventral anterior homeobox 1 (*VAX1*) gene has emerged as a significant genetic factor in the etiology of NSCL/P. Our study identified a significant association between the *VAX1* rs7078160 variant and NSCL/P risk in a Japanese population, supporting its role as a susceptibility locus for orofacial clefting. This finding is biologically plausible given *VAX1′*s fundamental role as a homeodomain transcription factor critical for embryonic development, particularly of the ventral forebrain and craniofacial midline structures, which provides strong biological plausibility for its involvement. Animal studies have demonstrated that mice homozygous for *VAX1* mutations exhibit craniofacial malformations, including cleft palate, while heterozygous mice appear normal [12]. Another study in animal models reported that *VAX1* knockout mice demonstrated that deficiency in *VAX1* function leads to severe craniofacial anomalies, including cleft palate, often linked to disruptions in Sonic hedgehog (Shh) signaling and impaired cellular proliferation in key developmental regions [13]. However, the animal models did not show detectable *VAX1* expression within the developing palate, and no differences in the overall anatomy or the rate of cellular proliferation in the palatal shelves were observed [12,13]. The association between *VAX1* rs7078160 and NSCL/P was initially robustly identified through genome-wide association studies (GWASs) and subsequently reinforced by large-scale meta-analyses that included diverse ancestral groups [6,30,31,32]. The results in our study are consistent with other replication studies in Southern Han Chinese, Western Han Chinese, Saudi Arabian, Estonian, Polish, and Mesoamerican populations [16,17,33,34,35,36]. In contrast, these were not consistently replicated in Brazilian, Slovak, or some Chinese studies, underscoring the variability of findings [37,38,39,40]. The frequency of the rs7078160 risk allele varies between populations, which may contribute to the discrepancies observed in different studies. The SNP rs7078160, located in an intergenic region near *VAX1* and *SHTN1* genes, likely contributes to NSCL/P risk by affecting gene regulation rather than directly altering protein structure [41]. This variant may influence transcription factor binding, enhancer activity, or epigenetic modifications that affect *VAX1* or *SHTN1* expression during critical periods of craniofacial development, potentially explaining its association with cleft risk [13,41]. The plausibility of *SHTN1* as a target is strengthened by independent studies that have also reported associations between other SNPs within the *SHTN1* gene itself and NSCL/P risk [42,43]. Recent studies have shown that such non-coding variants can influence gene expression over long distances by altering the activity of tissue-specific enhancer elements. It is plausible that rs7078160 lies within a distant-acting enhancer that physically interacts with the *VAX1* promoter during craniofacial development, a mechanism known to fine-tune gene expression critical for normal morphology [44]. Furthermore, the importance of regulatory variation affecting *VAX1* is underscored by other research, such as the recently described association of rs10787760 in the 3′ UTR of the *VAX1* gene with NSCL/P in a Chinese population [45].

Building upon our overall findings, sex-stratified analysis revealed a striking female-specific association for *VAX1* rs7078160 with NSCL/P: the A allele significantly increased risk in females but not males. This is noteworthy as, despite rs7078160 being an established NSCL/P risk locus, its sex-specific effects are rarely investigated [46]. Yet, exploring gene-by-sex (GxSex) interactions is vital for understanding sexually dimorphic traits like NSCL/P and can uncover novel sex-specific loci [47,48]. Our finding of a female-exclusive effect for rs7078160, contrasting with NSCL/P’s male predominance and supported by studies identifying autosomal loci with opposing sex effects, suggests differing underlying genetic mechanisms between sexes [3]. This sexual dimorphism could arise if sex-specific factors modulate *VAX1′*s crucial role in craniofacial development, including its Shh pathway interplay [12,13]. Plausible mechanisms include differential hormonal milieus, where rs7078160 might alter *VAX1′*s responsiveness to estrogens in females or androgens in males; sex-specific epigenetic modifications influencing *VAX1* pathway activity; or the rs7078160-A allele acting as a more potent risk factor in females under a multifactorial threshold model [49,50,51]. Elucidating these mechanisms is crucial for fully understanding NSCL/P etiology.

The *MAFB* gene region on chromosome 20q12, particularly SNP rs13041247, has been recognized as a susceptibility locus for NSCL/P. In our study, the C allele, specifically the CC genotype, showed a nominally protective effect against NSCLO. However, this association did not remain significant after Bonferroni correction, underlining the challenges of identifying robust associations in genetically complex traits such as NSCL/P. The initial GWAS by Beaty et al. (2010) first identified a strong association between rs13041247 and NSCL/P, with the C allele generally showing a protective effect [6]. Subsequent replication studies in diverse populations have produced inconsistent results. In East Asian cohorts, some studies in the Chinese Han population confirmed the associations, whereas broader meta-analyses of East Asian cohorts sometimes found non-significant associations [8,9]. In European populations, the association has been similarly variable. While large-scale meta-analyses detected modest associations or heterogeneity, others, such as a study in a Polish population, reported no significant association [8,52]. Similarly, investigations in admixed Brazilian populations reported mixed findings [53]. This variability highlights influences like population-specific genetic architecture, differing allele frequencies, gene–environment interactions, and study power. Our uncorrected findings in the NSCLO subgroup association align with this pattern of variable replication and potential subphenotype effects. Despite replication variability, *MAFB*’s role in NSCL/P pathogenesis is strongly supported by its biological function. As a transcription factor, *MAFB* is integral to orchestrating gene expression programs that are essential for proper differentiation and proliferation [3]. Furthermore, its expression has been detected in key structures for palate development in mouse models, specifically the palatal shelves and the medial edge epithelia (MEE) during their fusion [6]. Systematic reviews and meta-analyses consolidate evidence showing that *MAFB*’s expression in these tissues during critical embryonic periods is vital; its dysregulation may alter its expression or function and can disrupt these finely tuned developmental processes, such as the critical fusion of facial prominences, leading to the malformations characteristic of NSCL/P [54,55]. The intergenic rs13041247 likely has a regulatory function, potentially involving the alteration of transcription factor binding or the activity of distant-acting enhancer elements known to fine-tune craniofacial morphology, thereby influencing the expression levels or spatio-temporal patterning of *MAFB* itself [44,56]. Functional genomics data from resources like the ENCODE Project show that the region harboring rs13041247 contains chromatin marks consistent with enhancer activity in relevant cell types. Allelic variation at this SNP could therefore alter the binding of key transcription factors, thereby modulating *MAFB* expression levels during the critical window of palatal fusion [56]. Given *MAFB*’s established importance in controlling cellular behaviors fundamental to craniofacial development, such misexpression offers a plausible pathogenic mechanism for its contribution to NSCL/P risk.

Our research in a Japanese population revealed no genotypic or allelic association between the *WNT3* intronic SNP rs3809857 and susceptibility to NSCL/P or its subtypes. This null finding aligns with studies in other populations, such as a Polish cohort, where rs3809857 also showed no significant association with orofacial cleft risk, contributing to a complex understanding of this variant’s role [57]. However, the existing literature presents a mixed view. A meta-analysis by Wang et al. (2018) suggested the G allele of rs3809857 was linked to increased NSCL/P vulnerability in pooled populations, while an Iranian study indicated a protective effect for the heterozygous GT genotype [58,59]. Such discrepancies across studies highlight potential population-specific genetic effects, varying allele frequencies, or differing gene–environment interactions influencing NSCL/P susceptibility. The lack of significant association observed for *WNT3* rs3809857 (Table 3) may be partly attributed to limited statistical power, as the detectable odds ratio of 1.80 (Supplementary Table S1) suggests our study was powered to detect moderate to large effect sizes given the minor allele frequency of 0.2315. This highlights the need for larger sample sizes in future studies to detect smaller effects. Despite varied associations for rs3809857, *WNT3* is a strong biological candidate for NSCL/P. The Wnt signaling pathway, with *WNT3* as a critical ligand, indispensably regulates cell behaviors fundamental to craniofacial morphogenesis [60]. Lip and palate embryogenesis involves the highly orchestrated fusion of facial prominences [55]. *WNT3* signaling specifically coordinates these events, ensuring correct formation and merging of structures like the medial and lateral nasal prominences with maxillary prominences to form the upper lip and primary palate [21]. Disruption of this pathway, potentially from altered *WNT3* function or expression, can cause failed fusion and clefts [55]. If intronic rs3809857 influences NSCL/P risk, it likely does so by altering *WNT3* expression or splicing, thereby perturbing Wnt signaling during critical developmental windows [61].

Our study has several limitations: the molecular functions of the implicated *VAX1*, *MAFB*, and *WNT3*, particularly how *VAX1* rs7078160 mediates sex-specific effects, remain unelucidated; our analysis was confined to a single Japanese population; and a more comprehensive investigation of gene–gene and gene–environment interactions is warranted. Although our study focused exclusively on genetic associations, it is increasingly recognized that NSCL/P arises from complex gene–environment interactions. In the Japanese population, environmental exposures such as maternal smoking, alcohol consumption, and low folate or multivitamin intake during early pregnancy have been associated with elevated NSCL/P risk [62,63,64]. Evidence from genome-wide and family-based studies suggests that certain genetic variants may interact with maternal exposures to modulate susceptibility to NSCL/P [65,66]. Notably, IRF6 variants have shown interaction with maternal multivitamin use, underscoring the biological relevance of regulatory variants in folate-sensitive pathways [67]. Although gene–environment interaction analyses were beyond the scope of our current study, these findings highlight the need for integrative models that combine genetic and environmental data to better elucidate the multifactorial etiology of NSCL/P. Moreover, although we observed sex-specific associations in our stratified analyses, we did not perform formal statistical interaction tests to directly compare effect sizes between sexes. The odds ratios for the exploratory gene–gene interaction analysis were presented without adjustment for potential confounders such as sex, highlighting the need for replication in larger cohorts with fully adjusted models. Future research should prioritize (1) functional studies to determine these variants’ regulatory impact, especially in sex-dependent contexts; (2) replication of our findings for all three genes in larger, multi-ethnic cohorts; and (3) broader exploration of genetic and environmental contributors to NSCL/P’s sexual dimorphism to clarify its complex etiology. Future functional studies are essential to elucidate the molecular mechanisms underlying these associations. Specific approaches could include using CRISPR-Cas9-based enhancer assays in craniofacial progenitor cells to validate the regulatory potential of the non-coding SNPs rs7078160 and rs13041247, a technique that has been successfully used to functionally characterize other NSCL/P-associated loci [68]. Furthermore, to explore the biological basis of the observed sex-stratified association for *VAX1*, performing sex-specific transcriptomic or proteomic analyses in relevant craniofacial cell models would be a valuable next step. Such analyses are critical for identifying downstream pathways that are differentially affected in males and females, which is a known factor in human craniofacial development [69].

## 5. Conclusions

Our study demonstrates that *VAX1* rs7078160 significantly increases NSCL/P risk in the Japanese population, with stronger effects in females, while *MAFB* rs13041247 shows a weak protective effect specifically against NSCLO. Importantly, individuals carrying both *VAX1* rs7078160 A and *MAFB* rs13041247 C face nearly three times the risk of developing NSCLP and NSCL/P compared to those with only the *MAFB* variant, suggesting a synergistic interaction between these genes. In contrast, *WNT3* rs3809857 showed no significant association with orofacial clefts in this population. These findings contribute to our understanding of the population-specific genetic architecture of NSCL/P.

## Figures and Tables

**Table 1:** Characteristics of the study samples.

	NSCLO	NSCLP	NSCL/P	Control
**Male**	57	115	172	136
**Female**	58	80	138	172
**Total**	115	195	310	308

**Table 2:** Hardy–Weinberg equilibrium test and minor allele frequency for each SNP.

Chr	Gene	SNP	Allele	HWE *p*	MAF			
					Control	NSCLO	NSCLP	NSCL/P
10	*VAX1*	rs7078160	G > A	0.9987	0.459	0.600	0.579	0.587
20	*MAFB*	rs13041247	T > C	0.9742	0.406	0.296	0.410	0.368
17	*WNT3*	rs3809857	G > T	0.070	0.231	0.230	0.233	0.232

HWE *p*: Hardy–Weinberg equilibrium *p*-value.

**Table 3:** Association of SNPs for NSCL/P, two subtypes, and controls.

Genotype/Allele	Control (n = 308)	NSCLO (n = 115)	OR (95% CI)	*p* Value	NSCLP (n = 195)	OR (95% CI)	*p* Value	NSCL/P (n = 310)	OR (95% CI)	*p* Value
***VAX1*** **rs7078160**										
**GG**	90	22	Reference		32	Reference		54	Reference	
**AG**	153	48	1.28 (0.73–2.26)	0.388	100	1.84 (1.14–2.96)	0.012	148	1.61 (1.07–2.42)	0.021
**AA**	65	45	**2.83 (1.55–5.17)**	**0.0006**	63	**2.73 (1.60–4.64)**	**0.0002**	108	**2.77 (1.75–4.37)**	**<0.00001**
**Dominant model**	218	93	1.75 (1.03–2.95)	0.036	163	**2.10 (1.34–3.30)**	**0.0011**	256	**1.96 (1.33–2.87)**	**0.0005**
**Recessive model**	243	70	**2.40 (1.51–3.82)**	**0.0002**	132	**1.78 (1.19–2.68)**	**0.005**	202	1.58 (1.11–2.25)	0.011
**A**	283	138	**1.77 (1.30–2.40)**	**0.0003**	226	**1.62 (1.26–2.09)**	**0.0002**	364	**1.67 (1.34–2.1)**	**<0.00001**
**G**	333	92	Reference		164	Reference		256	Reference	
***MAFB*** **rs13041247**										
**TT**	108	45	Reference		65	Reference		110	Reference	
**CT**	149	52	0.84 (0.52–1.34)	0.459	100	1.12 (0.75–1.66)	0.592	152	1.00 (0.71–1.42)	0.993
**CC**	51	8	0.38 (0.17–0.86)	0.017 *	30	0.98 (0.57–1.69)	0.935	38	0.73 (0.44–1.20)	0.217
**Dominant model**	200	60	0.72 (0.45–1.13)	0.153	130	1.08 (0.74–1.58)	0.690	190	0.93 (0.67–1.30)	0.680
**Recessive model**	257	97	0.42 (0.19–0.91)	0.024 *	165	0.92 (0.56–1.50)	0.727	162	1.18 (0.74–1.88)	0.479
**C**	251	68	0.70 (0.50–0.97)	0.032 *	160	1.01 (0.78–1.31)	0.930	228	0.89 (0.71–1.12)	0.327
**T**	365	142	Reference		230	Reference		372	Reference	
***WNT3*** **rs3809857**										
**GG**	188	71	Reference		120	Reference		191	Reference	
**GT**	98	35	0.95 (0.59–1.52)	0.817	59	0.94 (0.63–1.40)	0.772	94	0.94 (0.67–1.34)	0.746
**TT**	22	9	1.08 (0.48–2.46)	0.849	16	1.14 (0.58–2.26)	0.708	25	1.12 (0.61–2.05)	0.718
**Dominant model**	120	44	0.97 (0.63–1.51)	0.895	75	0.98 (0.68–1.42)	0.911	119	0.98 (0.71–1.35)	0.884
**Recessive model**	298	106	1.15 (0.51–2.58)	0.734	179	1.21 (0.62–2.37)	0.575	285	1.19 (0.66–2.16)	0.567
**T**	142	53	1.02 (0.71–1.46)	0.911	91	1.04 (0.77–1.40)	0.811	144	1.03 (0.79–1.34)	0.820
**G**	474	177	Reference		299	Reference		476	Reference	

**Abbreviations**: CI: confidence interval, OR: odds ratio, dominant model: AA vs. Aa + aa, recessive model: aa vs. AA + Aa (a: minor allele). In bold are *p*-values that were significant after adjustment with Bonferroni correction in multiple tests (*p* ≤ 0.008). * Statistically significant at *p* < 0.05 level without Bonferroni correction.

**Table 4:** Association of SNPs with NSCL/P and two subtypes in females and males.

Genotype/Allele	Control (F/M)	NSCLO (F/M)	OR (95% CI) (F/M)	*p* Value (F/M)	NSCLP (F/M)	OR (95% CI) (F/M)	*p* Value (F/M)	NSCL/P (F/M)	OR (95% CI) (F/M)	*p* Value (F/M)
***VAX1*** **rs7078160**										
**GG**	56/34	13/9	Reference		14/18	Reference		27/27	Reference	
**AG**	86/67	18/30	0.90 (0.41–1.98) /1.69 (0.72–3.96)	0.797 /0.223	42/58	1.95 (0.98–3.90) /1.64 (0.84–3.20)	0.056 /0.149	60/88	1.45 (0.82–2.55) /1.65 (0.91–3.00)	0.199 /0.097
**AA**	30/35	27/18	**3.88 (1.75–8.60)** /1.94 (0.77–4.92)	**0.0006** /0.160	24/39	**3.2 (1.45–7.08)** /2.10 (1.01–4.37)	**0.0034** /0.045	51/57	**3.53 (1.85–6.71)** /2.05 (1.06–3.96)	**<0.00001** /0.031
**Dominant model**	116/102	45/48	1.67 (0.83–3.35) /1.78 (0.79–4.00)	0.145 /0.161	66/97	2.28 (1.18–4.40) /1.80 (0.95–3.39)	0.013 /0.069	111/145	1.98 (1.17–3.36) /1.79 (1.02–3.15)	0.010 /0.042
**Recessive model**	142/101	31/39	**4.12 (2.15–7.89)** /1.33 (0.68–2.62)	**<0.00001** /0.407	56/76	2.03 (1.09–3.77) /1.48 (0.86–2.55)	0.024 /0.159	87/115	**2.77 (1.64–4.69)** /1.43 (0.87–2.35)	**0.0001** /0.159
**A**	146/137	72/66	**2.22 (1.44–3.42)/** 1.35 (0.87–2.11)	**0.0003** /0.177	90/136	**1.74 (1.19–2.55)** /1.43 (1.00–2.03)	**0.0038** /0.05	162/202	**1.93 (1.40–2.66)** /1.40 (1.02–1.93)	**<0.00001** /0.039
**G**	198/135	44/48	Reference		70/94	Reference		114/142	Reference	
***MAFB*** **rs13041247**										
**TT**	61/47	23/22	Reference		21/44	Reference		44/66	Reference	
**CT**	79/70	27/25	0.91 (0.47–1.73) /0.76 (0.39–1.51)	0.767 /0.436	44/56	1.62 (0.87–3.00) /0.85 (0.50–1.47)	0.126 /0.569	71/81	1.25 (0.75–2.06) /0.82 (0.50–1.35)	0.391 /0.441
**CC**	32/19	8/10	0.66 (0.27–1.65) /1.12 (0.45–2.82)	0.375 /0.802	15/15	1.36 (0.62–3.00) /0.84 (0.38–1.86)	0.442 /0.673	23/25	0.73 (0.44–1.20) /0.94 (0.46–1.89)	0.217 /0.856
**Dominant model**	111/89	35/47	0.84 (0.45–1.54) /0.84 (0.44–1.59)	0.567 /0.593	59/71	1.54 (0.86–2.78) /0.85 (0.51–1.43)	0.146 /0.543	94/106	1.17 (0.73–1.89) /0.85 (0.53–1.35)	0.508 /0.490
**Recessive model**	140/117	50/47	0.7 (0.30–1.62) /1.31 (0.57–3.03)	0.403 /0.526	65/100	1.01 (0.51–1.99) /0.92 (0.45–1.91)	0.978 /0.831	115/147	0.88 (0.49–1.58) /1.05 (0.55–1.99)	0.657 /0.888
**C**	143/108	43/45	0.83 (0.54–1.28) /0.99 (0.63–1.55)	0.393 /0.966	74/86	1.21 (0.83–1.76) /0.91 (0.63–1.30)	0.323 /0.596	117/131	1.03 (0.75–1.43) /0.93 (0.67–1.29)	0.837 /0.681
**T**	201/164	73/69	Reference		86/144	Reference		159/213	Reference	
***WNT3*** **rs3809857**										
**GG**	107/81	32/39	Reference		49/71	Reference		81/110	Reference	
**GT**	52/46	19/16	1.22 (0.63–2.36) /0.72 (0.36–1.43)	0.550 /0.351	21/38	0.88 (0.48–1.62) /0.94 (0.55–1.61)	0.686 /0.828	40/54	1.02 (0.61–1.68) /0.86 (0.53–1.41)	0.950 /0.557
**TT**	13/9	7/2	1.80(0.66–4.89) /0.46 (0.10–2.24)	0.244 /0.501	10/6	1.68 (0.69–4.09) /0.76 (0.26–2.24)	0.250 /0.619	17/8	1.73 (0.79–3.76) /0.65 (0.24–1.77)	0.165 /0.400
**Dominant model**	65/55	26/18	1.34 (0.73–2.44) /0.68 (0.35–1.31)	0.343 /0.247	31/44	1.04 (0.60–1.80) /0.91 (0.55–1.52)	0.884 /0.724	57/62	1.16 (0.73–1.83) /0.83 (0.52–1.32)	0.529 /0.430
**Recessive model**	159/127	51/55	1.68 (0.63–4.44) /0.51 (0.11–2.45)	0.292 /0.512	70/109	1.75 (0.73–4.17) /0.78 (0.27–2.25)	0.205 /0.641	121/164	1.72 (0.80–3.67) /0.69 (0.26–1.83)	0.159 /0.453
**T**	78/64	33/20	1.36 (0.84–2.18) /0.69 (0.40–1.21)	0.209 /0.194	41/50	1.17 (0.76–1.82) /0.90 (0.59–1.37)	0.468 /0.633	74/70	1.25 (0.87–1.80) /0.83 (0.57–1.22)	0.234 /0.342
**G**	266/208	83/94	Reference		119/180	Reference		202/274	Reference	

**Abbreviations**: F: Females, M: Males, CI: confidence interval, OR: odds ratio, dominant model: AA vs Aa + aa, recessive model: aa vs AA + Aa (a: minor allele). In bold are *p*-values that were significant after adjustment with Bonferroni correction in multiple tests (*p* ≤ 0.004).

**Table 5:** Independent risk factors *VAX1* rs7078160 and *MAFB* rs13041247.

	Control	NSCLO	*p* Value	OR (95%CI)	NSCLP	*p* Value	OR (95%CI)	NSCL/P	*p* Value	OR (95%CI)
**Both *VAX1*** **and *MAFB***	138	58			113			171		
***VAX1*** **only**	80	35	0.8754	0.96 (0.58–1.59)	50	0.2199	1.31 (0.85–2.02)	85	0.4261	1.17 (0.80–1.70)
***MAFB*** **only**	62	12	0.0253	2.17 (1.09–4.33)	17	**0** **.** **0002**	**2** **.** **99** **(** **1** **.** **65–5** **.** **39** **)**	29	**0** **.** **00008**	**2** **.** **65** **(** **1** **.** **61–4** **.** **34** **)**

**Abbreviations:** CI: confidence interval, OR: odds ratio. In bold are *p*-values that were significant after adjustment with Bonferroni correction in multiple tests (*p* ≤ 0.008).

## Data Availability

The data that support the findings of this study are available from the corresponding author upon reasonable request.

## Supplementary Materials

The following supporting information can be downloaded at: https://www.mdpi.com/article/10.3390/genes16080862/s1, Table S1: Detectable Odds Ratios for SNPs Based on Statistical Power Analysis.
