# Supplementary material for: Association of VAX1, MAFB, WNT3 with Non-Syndromic Cleft Lip with or without Cleft Palate in a Japanese Population
Source: Genes (Basel). 2025 Jul 24;16(8):862. doi: 10.3390/genes16080862 (PMC12385629; doi:10.3390/genes16080862)
Supplement: Supplementary file 1 [file genes-16-00862-s001.zip › genes-3756186-supplementary.pdf]

**Supplementary Table S1** Detectable Odds Ratios for SNPs Based on Statistical Power Analysis

| | Average MAF | Detectable OR |
|---|---|---|
| **VAX1 rs7078160** | 0.523 | 1.64 |
| **MAFB rs13041247** | 0.387 | 1.66 |
| **WNT3 rs3809857** | 0.2351 | 1.80 |

Detectable odds ratios (ORs) were calculated using G*Power (version 3.1.9.7) with a two-tailed test, an $\alpha$ of 0.008 (Bonferroni-corrected for six tests), and a total sample size of 618 (310 cases and 308 controls). The effect size (w) was adjusted to achieve approximately 80% power (actual power ≈ 0.81) based on average minor allele frequencies (MAFs) derived from Table 2 (mean of case and control MAFs).
